# High prevalence of ST5-SCC*mec* II-t311 clone of methicillin-resistant *Staphylococcus aureus* isolated from bloodstream infections in East China

**DOI:** 10.1186/s12866-024-03232-5

**Published:** 2024-03-16

**Authors:** Qing Zhan, Gaoqin Teng, Weiwei Chen, Xiao Yu

**Affiliations:** 1 Infection Control Department, The First Affiliated Hospital, Zhejiang University School of Medicine, Hangzhou, 310003 People’s Republic of China; 2 Department of General Intensive Care Unit, The Second Affiliated Hospital of Zhejiang University School of Medicine, Hangzhou, 310003 People’s Republic of China; 3 Department of Clinical Laboratory Medicine, The First Affiliated Hospital, Zhejiang University School of Medicine, Hangzhou, 31000 People’s Republic of China; 4 NHC Key Laboratory of Pneumoconiosis, Shanxi Key Laboratory of Respiratory Diseases, Department of Pulmonary and Critical Care Medicine, The First Hospital of Shanxi Medical University, Taiyuan, 030001 People’s Republic of China

**Keywords:** Methicillin-resistant *staphylococcus aureus* (MRSA), ST5-SCC*mec* II-t311, Bloodstream infections (BSIs), Antimicrobial susceptibility, Virulence genes

## Abstract

**Objectives:**

Methicillin-resistant *Staphylococcus aureus* (MRSA) is a challenging global health threat, resulting in significant morbidity and mortality worldwide. This study aims to determine the molecular characteristics and antimicrobial susceptibility of 263 MRSA isolates in Zhejiang Province, east China.

**Methods:**

From 2014 to 2019, a total of 263 MRSA isolates from bloodstream infections (BSIs) were collected from 6 hospitals in 4 cities in Zhejiang province, east China. Antimicrobial susceptibility tests were conducted according to the guidelines set forth by the Clinical and Laboratory Standards Institute (CLSI). To characterize and analyze these isolates, multilocus sequence typing (MLST), staphylococcal cassette chromosome *mec* (SCC*mec*) typing, staphylococcal protein A (*spa*) typing and virulence genes gene profiles were performed.

**Results:**

The most predominant clone was ST5-SCC*mec* II-t311, which accounted for 41.8% (110/263), followed by ST59 (44/263, 16.7%). Compared with non-ST5-II-t311 isolates, ST5-II-t311 isolates were more resistant to erythromycin, tetracycline, levofloxacin, moxifloxacin, and ciprofloxacin, but more susceptible to clindamycin. Moreover, the rates of multidrug resistance were higher in ST5-II-t311 isolates compared to the non-ST5-II-t311 isolates. In comparison to the non-ST5-II-t311 isolates, ST5-II-t311 isolates showed no significant difference in virulence genes detected.

**Conclusions:**

MRSA ST5-II-t311 clone has become the most predominant clone in Zhejiang Province, east China and has higher rates of multidrug resistance than other isolates, that should be kept in mind when treating BSI. Moreover, MRSA ST59 clone shows an upward trend and has begun to spread into hospitals. Our findings highlight the importance of epidemiological studies of *S. aureus* carriage in the eastern region.

**Supplementary Information:**

The online version contains supplementary material available at 10.1186/s12866-024-03232-5.

## Introduction

*Staphylococcus aureus* is a Gram-positive pathogen, one of the major causes of hospital and community-acquired infections [1]. It has the potential to cause a wide range of diseases, and the severity of which can vary greatly. This is mainly related to its production of a variety of virulence factors, including staphylococcal enterotoxins (SEs), toxic shock syndrome toxin-1 (TSST-1), hemolysins, leukocidins, and exfoliative toxins, and immune-modulatory factors [2]. The most common problems are skin infections, and some of the most severe are bloodstream infections (BSIs), endocarditis, osteomyelitis, and necrotizing fasciitis [3]. These episodes are usually acute and resolve within a limited period but may lead to varying degrees of morbidity and mortality [4, 5].

Since the 1960s, several *S. aureus* clones have acquired the staphylococcal cassette chromosome *mec* (SCC*mec*), which is a mobile genetic element that confers resistance to methicillin and most β-lactam antibiotics [1, 6]. Methicillin-resistant *Staphylococcus aureus* (MRSA) has spread globally over the past few decades and has now become endemic in most hospitals and healthcare facilities in industrialized nations [7–10]. It is a leading cause of bacterial infections worldwide, and the length of hospital stay for MRSA reaches twice the length of other hospital stays, with a much higher financial burden on hospitals [5, 11]. Community-associated (CA) MRSA strains emerged in the late 1990s and have been increasingly reported in many developed countries around the world in the following decade [12]. By contrast with health-care-associated MRSA (HA-MRSA), CAMRSA was predominantly found in healthy individuals outside of hospital settings in the past [13]. However, over the last decade, there has been a blurring of the distinction between CA-MRSA and HA-MRSA.

While MRSA infections have a global occurrence, no uniform or single pandemic clone is circulating around the world. MRSA clones with distinct genetic traits and molecular characteristics predominate in different geographic regions. For instance, the USA300, an ST8-SCC*mec*-IVa MRSA clone, has been dominant in the United States, while ST80, ST59, and ST772 are prevalent in Europe and Asia respectively [14]. As a developing country, China’s healthcare system is unbalanced due to regional differences in economic development. Recent data from the CHINET surveillance system show that in 2021, the isolated rate of MRSA detection rates declined from previous years, but still accounted for 30% of all clinical staphylococcal isolates in China (www.chinets.com). A study confirmed that MRSA CC8-ST239 has lost its predominance and was replaced by CC59-ST59 and CC5 in china after 2015 [15]. Wu D et al. reported that MRSA ST5-II-t311 has become the predominant HA-MRSA clone in Hangzhou, Zhejiang Province from 2012 to 2013 [16]. Huang L, et al. reported the detection rates of MRSA stayed at moderate levels, and there was local transmission of MRSA from 2015 to 2017 [17]. Zhejiang Province is located in the east of China and has a good medical and health system. To control the spread of MRSA, the genotypic characteristics of MRSA clones should be understood not only at the national level but also at the regional level. Accordingly, the current study aimed to investigate the prevalence, antimicrobial resistance, molecular characteristics, and virulence genes associated with 263 MRSA isolates associated with BSIs between 2014 and 2019 from 6 hospitals in Zhejiang China.

## Materials and methods

### Collection and identification of MRSA isolates

**Fig. 1:**
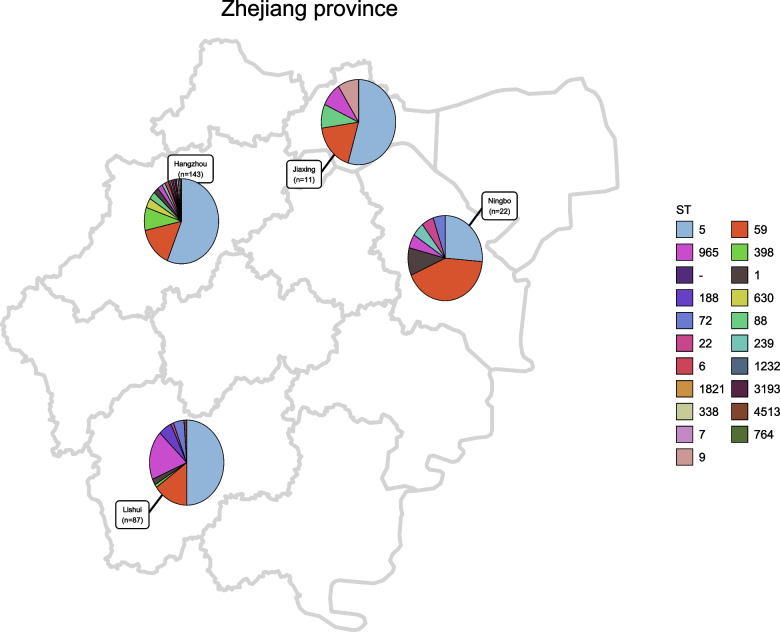
The clonal distribution of the 263 MRSA isolates in hospitals from 2014 to 2019 was included in this study. The map was created by using the corresponding map data with the R package ggplot2 (https://github.com/tidyverse/ggplot2). The map data were directly obtained from the R package maps v3.3.0 (https://github.com/adeckmyn/maps), which was imported from the Natural Earth data project (the 1:50 m world map, version 2.0, the latest version available in 2015)

From 2014 to 2019, a total of 263 MRSA isolates were cultured from the blood sample of patients with BSIs in 6 hospitals in Zhejiang province, China, including 143 from Hangzhou, 87 from Lishui, 22 from Ningbo, and 11 from Jiaxing (Fig. 1). According to the manufacturer’s instructions, these MRSA isolates were identified by Gram-staining and a VITEK-2 automated platform (bioMérieux, Marcy l’Etoile, France) as well as by additional biochemical testing that included Gram stain and catalase and coagulase activity with rabbit plasma. Cefoxitin screening was used for the initial screening and identification of MRSA, and the presence of the *mecA* gene was detected by PCR [18].

### Antimicrobial susceptibility testing

The antibiotic susceptibility profiles of all *S. aureus* isolates in the current study were performed using the bioMérieux VITEK2 system following manufacturer’s instructions. The following 14 antibiotics were tested: erythromycin, clindamycin, oxacillin, penicillin, tetracycline, vancomycin, rifampicin, ciprofloxacin, levofloxacin, amikacin, tigecycline, linezolid, daptomycin, and trimethoprim-sulfamethoxazole. The results were determined according to Clinical and Laboratory Standards Institute (CLSI) and European Committee on Antimicrobial Susceptibility Testing (EUCAST) recommendations, and interpreted based on the same guidelines [19, 20]. *S. aureus* ATCC 25923 was used as a standard strain to ensure quality control measures.

### DNA extraction and sequencing and detection of virulence genes

Genomic DNA (gDNA) of the 263 MRSA isolates was extracted using the Ezup Column Bacteria Genomic DNA Purification Kits (Sangon Biotech, Shanghai) according to the manufacturer’s instructions. The PCR amplification was carried out on a GeneAmp 9700 thermal cycler (Applied Biosystems, NY, United States). All MRSA isolates were screened for the following 30 staphylococcal virulence genes: staphylococcal enterotoxin genes (*sea*, *seb, sec*, *sed*, *see*, *seg*, *seh*, *sei*, *sej*, *sel*, *sem*, *sen*, *seo*, *sep*, *seq*), fibronectin-binding protein A (*fnbA*), toxic shock syndrome toxin (*tsst1*), exfoliative toxin genes (*eta*, *etb*), leukocidin (*lukF-PV*, *lukDE*), hemolysin genes (*hla*, *hlb*, *hlg*, *hly*), and adhesin genes (*clfA*, *icaA*, *sdrC*, *sdrD*, and *sdrE*), as previously described [21–23].

### Multi-locus sequence typing (MLST)

All MRSA isolates were performed with multilocus sequence typing (MLST) by using primers targeting seven standard housekeeping genes (*arcC*, *aroE*, *gmk*, *glpF*, *pta*, *tpi* and *yqil*) listed on the PubMLST website (https://pubmlst.org/organisms/staphylococcus-aureus/primers) according to previously published methods, and the MLST database was used to determine the sequence types (STs) [24].

### *Spa* typing

The X region of the *spa* gene of each MRSA isolate was amplified by PCR as described previously. *Spa* typing was obtained by submitting the data to the *S. aureus spa* type database [18, 25].

### Staphylococcus chromosomal cassette *mec* (SCC*mec*) typing

SCC*mec* typing was performed on all MRSA isolates as described previously, which is based on a set of multiplex PCR reactions with 14 primers specific for SCC*mec* types and sub-types. SCC*mec* types I–V were assigned based on a combination of the cassette chromosome recombinase (ccr) type and *mec* class, and some MRSA isolates that could not be assigned to any of the expected types were defined as non-type (NT) [26].

### Statistical analysis

All data analyses were analyzed using the SPSS statistical software package (version 19; IBM SPSS Statistics). The chi-square test was used for categorical variables. *P* values of < 0.05 were considered statistically significant.

## Results

### Phenotypic and molecular characteristics

**Fig. 2:**
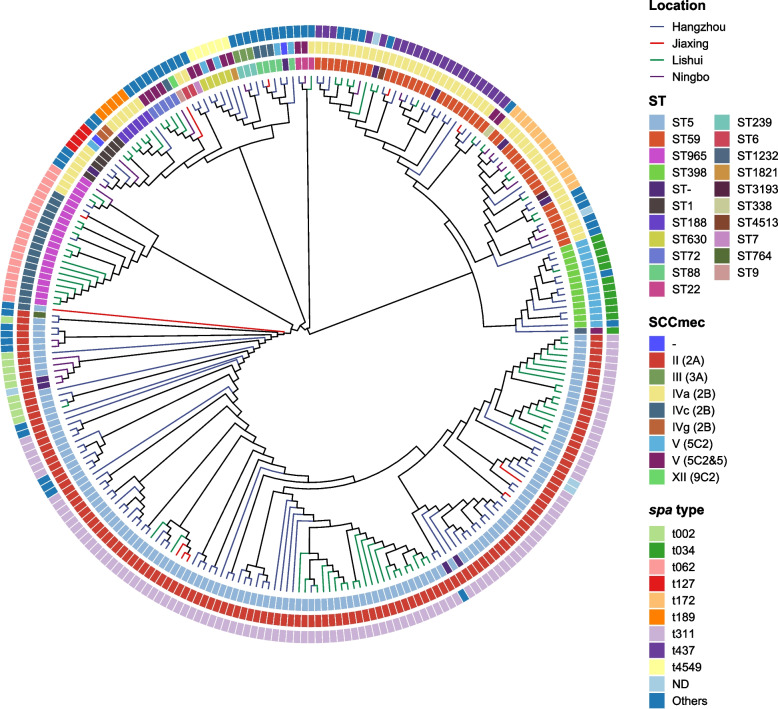
Phylogenetic tree of all 263 MRSA. Locations are distinguished by different colors with lines. ST types, Spa types, and SCC*mec* types are color-coded in the inner rings

As show in Table 1 and Fig. 2, the 263 MRSA isolates was assigned into 20 sequence types, 49 *spa* types, and 12 SCC*mec* types. The evolutionary and genetic diversity of all MRSA isolates was analyzed by MLST and *spa* typing. Twenty distinct STs were identified within the 263 MRSA isolates, of which ST5 was identified as the most prevalent ST type, accounting for 49.8% (131/263), followed by ST59 (44/263, 16.7%), ST965 (21/263, 8.0%), ST398 (13/263, 4.9%), ST1 (7, 2.7%) ST188 (6, 2.3%), ST88 (5, 1.9%), ST630 (5, 1.9%), ST72 (5, 1.9%), ST239 (3, 1.1%) and ST22 (3, 1.1%). Each of the remaining STs accounted for only one or two isolates, and the STs for 10 isolates were not identified. As show in Fig. 1, ST5 was the most prevalent ST type in Lishui, Hangzhou and Jiaxin, and ST59 in Ningbo. These MRSA isolates were classified into 49 *spa* types, and 5 isolates had unknown *spa* types. The majority of MRSA isolates belonged to one major *spa* type, t311 (111/263, 42.2%), followed by t062 (19, 7.2%), t437 (19, 7.2%), t172 (14, 5.3%), t034 (12, 4.6%), t002 (10, 3.8%), t4549 (6, 2.3%), t189 (5, 1.9%), t2460 (4, 1.5%), t127 (4, 1.5%), t437 (4, 1.5%) and t163 (4, 1.5%). The rest of the *spa* types represented by fewer than three isolates. By SCC*mec* typing, five types (types II, III, IV, V and XII) were found among 263 MRSA isolates, 2 isolates were classified as nontypable for SCC*mec* typing. The most common was type II (135/263, 51.3%), followed by type IV (90, 34.2%), type V (32, 12.2%), type III (3, 1.1%) and type XII (1, 0.4%).

**Table 1:** Molecular characteristics of 263 MRSA isolates

MLST (*n* ^a^)	SCC*mec* (*n*)	Spa (*n*)
ST5(131)	II(130)	t311(110), t002(9), t2460(4), t1251(3), t2731(1), t3557(1), None(2)
	IV(1)	t1560(1)
ST59(44)	IVa(43)	t437(20), t172(11), t163(4), t3523(2), t441(2), t16634(1), t6596(1), None(2)
	V(1)	t437(1)
ST965(21)	IV(21)	t062(20), t1399(1)
ST398(13)	V(13)	t034(11), t011(1), t1456(1)
ST1(7)	IV(5)	t114(2), t127(2), t1908(1)
	V(1), None(1)	t127(2)
ST188(6)	IV(6)	t189(5), t3887(1)
ST88(5)	IV(3)	t2310(2), t15074(1)
	V(2)	t7637(1), t2526(1)
ST630(5)	V(5)	t549(5)
ST72(5)	V(4)	t148(3), t4100(1)
	IV(1)	t2461(1)
ST22(3)	V(2)	t309(2)
	IV(1)	t032(1)
ST239(3)	III(3)	t030(1), t421(1), t037(1)
ST6(2)	IV(2)	t304(2)
ST3193(1)	IV(1)	t172(1)
ST338(1)	V(1)	t13774(1)
ST764(1)	II(1)	t045(1)
ST4513(1)	IV(1)	t8886(1)
ST9(1)	XII(1)	t899(1)
ST7(1)	V(1)	t091(1)
ST1821(1)	V(1)	t4549(1)
ST1232(1)	V(1)	t034(1)
ST-(10)	II(4)	t311(1), t002(1), None(1), t3235(1)
	IV(5)	t172(2), t693(1), t437(2), t437(1), t437(1)
	NA(5)	t637(1)

*MLST* multilocus sequence typing, *SCCmec* staphylococcus chromosomal cassette mec, *spa* staphylococcal protein A, *NT* non-typeable

^a^ *n* number of isolates in each type

Among the 263 MRSA isolates, ST5-SCC*mec* II-t311 isolates accounted for 41.8% (110/263), being the predominant clone, followed by ST59-SCC*mec* IVa-t437 (21/263, 8.0%) and ST398-V-t034 (11/263, 4.2%), as determined by *spa* typing, MLST, and SCC*mec* typing (Fig. 2).

### Antimicrobial susceptibility testing

All MRSA isolates were resistant to penicillin and oxacillin, and no MRSA isolates were resistant to vancomycin, daptomycin and linezolid. The antimicrobial susceptibility test results for the other ten antibiotics are shown in Table 2, and the antimicrobial susceptibility test results of MRSA ST5 and ST59 are shown in Table S2. Most of the MRSA isolates exhibited multiple antibiotic-resistance profiles. Specifically, more than 50% of isolates were resistant to four of the ten remaining antibiotics, namely erythromycin (81.4%), ciprofloxacin (57.0%), levofloxacin (63.9%), moxifloxacin (61.2%). The resistance rates to other antibiotics tested were 46.8% to clindamycin, 1.5% to trimethoprim-sulfamethoxazole, 36.99% to tetracycline, 1.4% to rifampicin 0.8% to amikacin and 3.4% to tigecycline.

**Table 2:** The antibiotic resistance rates of 263 MRSA isolates

Antibiotics^a^	Total (*n* ^b^ = 263); R^c^	ST5-II-t311 (*n* = 110); R	Other isolates (*n* = 153); R	*P*-value^d^
OXA	263; 100.0%	110; 100.0%	153; 100%	> 0.05
PEN	263; 100.0%	110; 100.0%	153; 100%	> 0.05
ERY	214; 81.4%	104; 94.5%	110; 71.9%	< 0.05
CLN	123; 46.8%	40; 36.4%	83; 54.2%	< 0.05
SXT	4; 1.5%	1; 0.9%	3; 2.0%	> 0.05
TCY	97; 36.9%	61; 55.5%	36; 23.5%	< 0.05
VAN	0; 0.0%	0; 0.0%	0; 0.0%	> 0.05
RIF	3; 1.4%	0; 0.0%	3; 2.0%	> 0.05
CIP	150; 57.0%	85; 77.3%	65; 42.5%	< 0.05
LVX	168; 63.9%	104; 94.5%	64; 41.8%	< 0.05
MFX	161; 61.2%	105; 95.5%	56; 36.6%	< 0.05
AMK	2; 0.8%	1; 0.9%	1; 0.7%	> 0.05
TGC	9; 3.4%	6; 5.5%	3;2.0%	> 0.05
LNZ	0; 0.0%	0; 0.0%	0; 0.0%	> 0.05
DAP	0; 0.0%	0; 0.0%	0; 0.0%	> 0.05

^a^Oxacillin (OXA), penicillin (PEN), erythromycin (ERY), clindamycin (CLN), trimethoprim-sulfamethoxazole (SXT), tetracycline (TCY), vancomycin (VAN), rifampicin (RIF), ciprofloxacin (CIP), levofloxacin (LVX), moxifloxacin (MFX), amikacin (AMK), tigecycline (TGC), linezolid (LNZ), daptomycin (DAP)

^b^ *n* number of isolates in each type

^c^ *R* resistance

^d^The resistance rates of antimicrobials among ST5-II-t311 isolates were compared to those among Non-ST5-II-t311 isolates. *P* < 0.05 was considered statistically significant

Compared with non-ST5-II-t311 isolates, ST5-II-t311 isolates were more resistant to erythromycin, tetracycline, levofloxacin ciprofloxacin and moxifloxacin, but more susceptible to clindamycin (*P* <  0.05). In addition, ST5-II-t311 isolates had higher rates of multidrug resistance than the non-ST5-II-t311 isolates (Table 2).

Besides, compared with ST5 isolates, ST59 isolates were more susceptible to erythromycin, tetracycline, levofloxacin, ciprofloxacin and moxifloxacin, but more resistant to clindamycin and all ST59 isolates were susceptible to trimethoprim-sulfamethoxazole and rifampicin (Table S2).

### Virulence gene profiles

The distribution of 30 representative virulence genes varied among the 263 BSI MRSA isolates according to MLST types (Table 3). In our study, the toxin genes *etb* and *see* were not discovered among all MRSA isolates, and all isolates simultaneously harbored at least four virulence genes (*icaA*, *hlb*, *hlg* and *eta*). 59.3% (156/263) isolates harbored ≥19 tested virulence genes, among which one isolate harbored 23 genes, 98 isolates harbored 22 genes, 21 isolates harbored 21 genes, 11 isolates harbored 20 genes, 18 isolates harbored 19 genes.

**Table 3:** Frequencies of virulence genes among 263 MRSA isolates

Virulence gene	Total (*n* ^a^ = 263); P^b^	ST5 (*n* = 133); *P*	ST59 (*n* = 44); P	ST965 (*n* = 21); P	ST398 (*n* = 13); *P*
lukF-PV	15; 5.7%	3; 2.3%	3; 6.8%	1; 4.8%	1; 7.7%
lukDE	188; 71.5%	95; 72.5%	30; 68.2%	16; 76.2%	12; 92.3%
tsst-1	137; 52.1%	75; 57.3	19; 43.2%	11; 52.4%	7; 53.8%
clfA	244; 92.8%	122; 93.1%	40; 90.9%	21; 100.0%	12; 92.3%
icaA	263; 100.0%	131; 100.0%	44; 100.0%	21; 100.0%	13; 100.0%
hly/hla	262; 99.6%	130; 99.2%	44; 100.0%	21; 100.0%	13; 100.0%
hlb/hlg	263; 100.0%	131; 10%	44; 100.0%	21;100.0%	13; 100.0%
sea	150; 57.0%	76; 58.0%	25; 56.8%	11; 52.4%	8; 61.5%
seb	47; 17.9%	24; 18.3%	9; 20.5%	2; 9.5%	0; 0.0%
sec	140; 53.2%	76; 58.0%	19; 43.2%	13; 61.9%	7; 53.8%
sed	1; 0.4%	1; 0.8%	0; 0.0%	0; 0.0%	0; 0.0%
seg	165; 62.7%	83; 63.4%	26; 59.1%	13; 61.9%	8; 61.5%
seh	8; 3.0%	2; 1.5%	2; 4.5%	2; 9.5%	1; 7.7%
sei	165; 62.7%	83; 63.4%	26; 59.1%	13; 61.9%	8; 61.5%
sej	1; 0.4%	1; 0.8%	0; 0.0%	0; 0.0%	0; 0.0%
sel	140; 53.2%	76; 58.0%	19; 43.2%	13; 61.9%	7; 53.8%
sem/sen	165; 62.7%	83; 63.4%	26; 59.1%	13; 61.9%	8; 61.5%
seo	166; 63.1%	83; 63.4%	26; 59.1%	13; 61.9%	9; 69.2
sep	8; 3.0%	4;3.1%	2; 4.5%	0; 0.0%	1; 7.7%
seq	53; 20.2%	27; 20.6%	11; 25.0%	4; 19.0%	0; 0.0%
sdrC	256; 97.3%	129; 98.5%	43; 97.7%	20; 95.2%	12; 92.3%
sdrD	200; 76.0%	100; 76.3%	34; 77.3%	15; 71.4%	12; 92.3%
sdrE	235; 89.4%	118; 90.1%	39; 88.6%	17; 81.0%	13; 100%
eta	263; 100.0%	131; 100%	44; 100%	21; 100.0%	13; 100%
see/etb	0; 0.0%	0; 0.0%	0; 0.0%	0; 0.0%	0; 0.0%
fnbA	260; 98.9%	130; 99.2%	43; 97.7%	21; 100.0%	12; 92.3%

^a^ *n* number of isolates in each type

^b^ *P* positive rate

The adhesion genes were existed in most MRSA isolates, among which 100% carried the *icaA* genes, 92.8% carried *clfA* genes, 97.3% harbored *sdrC* genes, and 89.4% carried *sdrE* genes. Fifteen classical enterotoxin genes (*sea*, *seb*, *sec*, *sed*, *see*, *seg*, *seh*, *sei*, *sej*, *sel*, *sem*, *sen*, *seo*, *sep* and *seq*) were detected within 263 MRSA isolates (Table 3). The positivity rates for *sed* (0.4%), *seh* (3.0%), *sej* (0.4%) and *sep* (3.0%) among all MRSA isolates were low, most of the remaining enterotoxin genes have a positive rate of more than 50% except for *seb* (17.9%) and *seq* (20.2%). The toxin-encoding genes detected were *hly/hla* (100%), *lukD/E* (71.5%), *hlg/hlb* (100%), *tsst1* (52.1%) and *fnbA* (98.9%), and fifteen isolates (5.7%) carried *lukS/F-pv* genes.

As show in Table 3, the distribution of enterotoxin genes is related to ST type. The genes *seq* and *seb* were not detected in ST398 but in ST5, ST65 and ST965, and the genes *sed* were not detected in ST398, ST65 and ST965 but in ST5. The *sep* genes were more frequently detected in ST5, ST65 and ST965 than in ST72 (*P* <  0.05). The *sej* genes were only detected in ST5 but not in ST59, ST965 and ST398. The carrier rate of most virulence genes in ST59 was slightly lower than that in ST5, but it was not statistically significant (*p* > 0.05). In comparison to the non-ST5-II-t311 isolates, ST5-II-t311 isolates showed no not statistically significant in virulence genes detected (*p* > 0.05) (Table 4).

**Table 4:** Frequencies of virulence genes among ST5-II-t311 isolates and non- ST5-II-t311 isolates

Virulence gene	Total (*n* ^a^ = 263); P^b^	ST5-II-t311 isolates (*n* = 110); P	Other isolates (*n* = 153); P	*P*-value^c^
lukF-PV	15; 5.7%	3; 2.7%	12; 7.8%	> 0.05
lukDE	188; 71.5%	80; 72.7%	108; 70.6%	> 0.05
tsst-1	137; 52.1%	64; 58.2%	73; 47.7%	> 0.05
clfA	244; 92.8%	104; 94.5%	140; 91.5%	> 0.05
icaA	263; 100.0%	110; 100.0%	153; 100.0%	> 0.05
hly/hla	262; 99.6%	109; 99.1%	153; 100.0%	> 0.05
hlb/hlg	263; 100.0%	110; 100.0%	153; 100.0%	> 0.05
sea	150; 57.0%	65; 59.1%	85; 55.6%	> 0.05
seb	47; 17.9%	21; 19.1%	26; 17.0%	> 0.05
sec	140; 53.2%	65; 59.1%	75; 49.0%	> 0.05
sed	1; 0.4%	1; 0.9%	0; 0.0%	> 0.05
seg	165; 62.7%	71; 64.5%	94; 61.4%	> 0.05
seh	8; 3.0%	2; 1.8%	6; 3.9%	> 0.05
sei	165; 62.7%	71; 64.5%	94; 61.4%	> 0.05
sej	1; 0.4%	1; 0.9%	0; 0.0%	> 0.05
sel	140; 53.2%	65; 59.1%	75; 49.0%	> 0.05
sem/sen	165; 62.7%	71; 64.5%	94; 61.4%	> 0.05
seo	166; 63.1%	71; 64.5%	95; 62.1%	> 0.05
sep	8; 3.0%	3; 2.7%	5; 3.3%	> 0.05
seq	53; 20.2%	23; 20.9%	30; 19.6%	> 0.05
sdrC	256; 97.3%	109; 99.1%	147; 96.1%	> 0.05
sdrD	200; 76.0%	88; 80.0%	112; 73.2%	> 0.05
sdrE	235; 89.4%	100; 90.9%	135; 88.2%	> 0.05
eta	263; 100.0%	110; 100.0%	153; 100.0%	> 0.05
see/etb	0; 0.0%	0; 0.0%	0; 0.0%	> 0.05
fnbA	260; 98.9%	110; 100.0%	153; 100.0%	> 0.05

^a^ *n* number of isolates in each type

^b^ *P* positive rate

^c^The positive rate of Virulence genes among ST5-II-t311 isolates were compared to those among Non-ST5-II-t311 isolates. *P* < 0.05 was considered statistically significant

## Discussion

Bloodstream infections due to MRSA are mostly connected with high morbidity and mortality [27]. It is a challenging global health threat, which has a high potential for adaptation, enabling its successful survival in diverse environments and the continuous emergence of new lineages that exhibit rapid epidemiological dissemination. Previous studies show that the prevalence of MRSA varies substantially worldwide, and also differs greatly from provinces and cities in most countries [28]. It is essential to investigate the current epidemiological situation in various regions. Zhejiang, an economically advanced province along the east coast of China, with a robust healthcare system. We analyzed the molecular and epidemiological characteristics of 263 MRSA isolates associated with BSIs over 6 years in Zhejiang.

The major epidemic MRSA clones are generally related to specific geographical regions. Since 2010, MRSA ST59 has become the dominant clone in most hospitals in China [29]. The prevalence for MRSA varies geographically in such a vast country. Many reports were indicating that ST5 was the dominant MRSA type in eastern China [16, 30]. This is similar to our results, the MRSA ST5-SCC*mec* II clone was the most epidemic lineage in Zhejiang, followed by ST59-SCC*mec* IV. Among 263 MRSA isolates, *spa*-t311 clone was accounting for 42.2% (111/263), and 110 were ST5-II-t311. These results indicated that MRSA ST5-II-t311 clone was the predominant clone in Zhejiang. ST5-MRSA-II isolates, or the New York Japanese strain, typically manifest as spa t002, t003, or (in Ireland) t045. In this study, ST5-SCC*mec* II-t311 was derived from ST5-SCC*mec* II-t002, which differed between the New York/Japan clones [31, 32]. Nevertheless, the genetic mechanism underlying the successful spread of ST5 remains elusive. Some studies believed that ST5 strains carried more efflux pump genes, thus surviving in these hospitals with better hand hygiene and were transmitted through hands [33, 34]. Some studies have affirmed the role of SCC*mec* elements for being transmitted and multiplied by helping their hosts to cope with antibiotic compounds, and they also considered that a polyphyletic origin by multiple and independent transfers of SCC*mec* II elements to various CC5-MSSA precursor strain [31, 32]. In our study, ST5-II-t311 isolates showed a higher rate of multidrug resistance compared to non-ST5-II-T311 isolates. Thus, its prevalence may be due to its possible acquisition of other resistance genes.

In addition, previous studies indicated that the pandemic clone in certain geographic regions has been evolving. In the United States, ST8-IV (USA300) was the most dominant CA-MRSA strain [28]. Additionally, Joshua T. Smith et al. reported that the MRSA population responsible for BSIs in the Americas was predominantly influenced by the clonal expansion of two distinct lineages ST8 and ST5 [35]. A study by Norihito Kaku et al. indicated that ST8-IV and ST1-IV have replaced ST5-II in MRSA BSIs in Japan [36]. In South Korea, ST5 and ST72 have been identified as the predominant HA- and CA-MRSA, respectively. In China, local clonal expansion followed by substitution of contemporary lineages was reported in *S. aureus* ST239 by ST59, while in Hungary, this phenomenon was observed with ST5 and ST228 replacing ST239 [29, 37, 38]. In our results, ST59 accounted for 16.7%, ranked second, and most of them are ST59-SCC*mec* IV. Compared to types II, MRSA-IV and V clones harbored smaller SCC*mec* elements [39]. A previous study has demonstrated that smaller SCC*mec* cassettes may benefit its transmission by reducing the fitness burden [40]. These results showed that ST59 MRSA has begun to spread into hospitals in Zhejiang, and it may show an upward trend. Wang B et al. also confirmed CC59-ST59 and CC5 have been the most predominant clone in 7 provinces and municipalities of China between 2014 and 2020, but multiple CC5 clones, including ST5-t311- II, ST5-t2460-II, ST764-t1084-II and ST764-t002-II, were alternatively predominant many times over the past seven year [15]. However, in this study, ST5-II-t311 was the most predominant every year from 2014 to 2019 (Table S3). This is mainly due to the fact that most of the CC5 strains in the study of Wang B et al. were isolated from sputum specimens, while all MRSA isolates in this study were isolated from blood specimen sine MRSA is the second leading cause of BSI in Zhejiang province [41].

Most of the MRSA isolates in this study showed multidrug resistance but remained susceptible to vancomycin, linezolid and daptomycin. Y. Jian ET AL. et al. reported that MRSA ST5 clones could be more tolerant under the threat of antibacterial agents by NGS data and vitro susceptibility tests [30]. In the current study, ST5-II-t311 isolates were more resistant to erythromycin, tetracycline, levofloxacin, ciprofloxacin, and moxifloxacin than non-ST5-II-t311 isolates (*P* < 0.05). In addition, ST5-II-t311 isolates exhibited higher rates of multidrug resistance in comparison to the non-ST5-II-t311 isolates. It may provide an explanation for the prevalence of ST5-II-t311 isolates in recent years, with multidrug resistance making these isolates more competitive in hospital settings.

The pathogenicity of *S. aureus* is mainly related to the presence of multiple virulence factors, including staphylococcal enterotoxins (SEs), leucocidins, hemolysins, toxic shock syndrome toxin-1 (TSST-1), and exfoliative toxins, and immune-modulatory factors [42]. In our study, adhesion genes were present in most MRSA isolates; 100% carried the *icaA* genes, 92.8% carried *clfA* genes, 97.3% harbored *sdrC* genes, and 89.4% carried *sdrE* genes. Fifteen classical enterotoxin genes (*sea*, *seb*, *sec, sed*, *see, seg*, *seh*, *sei*, *sej*, *sel*, *sem*, *sen, seo*, *sep* and *seq*) were detected within 263 MRSA isolates. The *pvl* gene has previously been strongly associated with CA-MRSA and is linked to skin and soft tissue infections (SSTIs) in many studies [43]. The prevalence of *pvl* genes in MRSA isolates (5.7%) was low in our study, probably due to the fact that the samples were all from bacteremia. Besides the distribution of enterotoxin genes were reported to be related to specific molecule types [44]. In our results, the SEs genes *seb*, *sed, sej* and *seq* were not founded in ST398, sed and *sej* were only detected in ST5. The *sep* genes were more frequently detected in ST5, ST65 and ST965 than in ST72. However, in comparison to the non-ST5-II-t311 isolates, ST5-II-t311 isolates showed no significant difference in virulence genes detected. These results suggest that various molecular types are associated with different virulence profiles, but not necessarily the crux of their high prevalence.

However, this study has some limitations. Firstly, as retrospective research, it is not possible to confirm whether different genotypes are related to the clinical outcome due to incomplete clinical information. Secondly, all MRSA isolates were isolated from bloodstream infections, therefore, a single-center sample may not be representative of MRSA isolated from various samples. Therefore, it is important to exercise caution when interpreting our findings.

In conclusion, we analyzed the evolution in the molecular and epidemiological characteristics of 263 MRSA isolates associated with bloodstream infections over a time span of 6 years in Zhejiang Province, China. In our results, ST5-II-t311 MRSA was the predominant clone, and the multi-drug resistance rate of MRSA ST5-II-t311 isolates was higher than that of other isolates. Moreover, ST59 MRSA shows an upward trend and has begun to spread into several hospitals in our study. It is necessary to continuously monitor the trends in MRSA prevalence.

### Supplementary Information

**Supplementary material 1.**

**Supplementary material 2.**

**Supplementary material 3.**

## Data Availability

The datasets used during the current study are available from the corresponding author upon reasonable request. Most of the data is included in this article.

## References

[1] Tong S (2015). Staphylococcus aureus infections: epidemiology, pathophysiology, clinical manifestations, and management. Clin Microbiol Rev.

[2] Kuehl R (2020). When antibiotics fail: a clinical and microbiological perspective on antibiotic tolerance and persistence of Staphylococcus aureus. J Antimicrob Chemother.

[3] Tacconelli E, Tumbarello M, Cauda R (1998). Staphylococcus aureus infections. N Engl J Med.

[4] Whitby M, McLaws M, Berry G (2001). Risk of death from methicillin-resistant Staphylococcus aureus bacteraemia: a meta-analysis. Med J Aust.

[5] Gould I (2010). Costs of healthcare-associated methicillin-resistant Staphylococcus aureus and its control. Clinical microbiology and infection : the official publication of the European Society of Clinical Microbiology and Infectious Diseases.

[6] Eriksen K (1961). “Celbenin”-resistant staphylococci. Ugeskr Laeger.

[7] Turner N (2019). Methicillin-resistant Staphylococcus aureus: an overview of basic and clinical research. Nat Rev Microbiol.

[8] David M (2014). Staphylococcus aureus bacteremia at 5 US academic medical centers, 2008-2011: significant geographic variation in community-onset infections. Clinical infectious diseases : an official publication of the Infectious Diseases Society of America.

[9] David M (2015). Pediatric Staphylococcus aureus isolate genotypes and infections from the Dawn of the community-associated methicillin-Resistant S. Aureus epidemic era in Chicago, 1994 to 1997. J Clin Microbiol.

[10] Chen Y (2022). Epidemiology, evolution and cryptic susceptibility of methicillin-resistant Staphylococcus aureus in China: a whole-genome-based survey. Clinical microbiology and infection : the official publication of the European Society of Clinical Microbiology and Infectious Diseases.

[11] Nelson R (2019). Methicillin-resistant Staphylococcus aureus colonization and pre- and post-hospital discharge infection risk. Clinical infectious diseases : an official publication of the Infectious Diseases Society of America.

[12] Elston D (2007). Community-acquired methicillin-resistant Staphylococcus aureus. J Am Acad Dermatol.

[13] Herold B (1998). Community-acquired methicillin-resistant Staphylococcus aureus in children with no identified predisposing risk. JAMA.

[14] David M, Daum R (2010). Community-associated methicillin-resistant Staphylococcus aureus: epidemiology and clinical consequences of an emerging epidemic. Clin Microbiol Rev.

[15] Wang B (2022). Staphylococcus aureusMethicillin-resistant in China: a multicentre longitudinal study and whole-genome sequencing. Emerging microbes & infections.

[16] Wu D (2018). Predominance of ST5-II-t311 clone among healthcare-associated methicillin-resistant Staphylococcus aureus isolates recovered from Zhejiang, China. International journal of infectious diseases : IJID : official publication of the International Society for Infectious Diseases.

[17] Huang L (2019). Staphylococcus aureusEpidemiology and risk factors of methicillin-resistant and vancomycin-resistant infections in Zhejiang China from 2015 to 2017. Antimicrob Resist Infect Control.

[18] Kondo Y (2007). Combination of multiplex PCRs for staphylococcal cassette chromosome mec type assignment: rapid identification system for mec, ccr, and major differences in junkyard regions. Antimicrob Agents Chemother.

[19] CLSI (2021). Performance standards for antimicrobial susceptibility testing, M100, 31st ed. Clinical and Laboratory Standards Institute.

[20] EUCAST (2021). Breakpoint tables for interpretation of MICs and zone diameters, version 11.0.

[21] Omoe K (2002). Detection of seg, seh, and sei genes in Staphylococcus aureus isolates and determination of the enterotoxin productivities of S. Aureus isolates harboring seg, seh, or sei genes. J Clin Microbiol.

[22] Lina G (1999). Involvement of Panton-valentine leukocidin-producing Staphylococcus aureus in primary skin infections and pneumonia. Clinical infectious diseases : an official publication of the Infectious Diseases Society of America.

[23] Warner J, Onderdonk A (2004). Diversity of toxic shock syndrome toxin 1-positive Staphylococcus aureus isolates. Appl Environ Microbiol.

[24] Enright M (2000). Multilocus sequence typing for characterization of methicillin-resistant and methicillin-susceptible clones of Staphylococcus aureus. J Clin Microbiol.

[25] Koreen L (2004). Spa typing method for discriminating among Staphylococcus aureus isolates: implications for use of a single marker to detect genetic micro- and macrovariation. J Clin Microbiol.

[26] Zhang K (2005). Novel multiplex PCR assay for characterization and concomitant subtyping of staphylococcal cassette chromosome mec types I to V in methicillin-resistant Staphylococcus aureus. J Clin Microbiol.

[27] Seybold U (2006). Emergence of community-associated methicillin-resistant Staphylococcus aureus USA300 genotype as a major cause of health care-associated blood stream infections. Clinical infectious diseases : an official publication of the Infectious Diseases Society of America.

[28] Lakhundi S, Zhang K. Methicillin-resistant Staphylococcus aureus: molecular characterization, evolution, and epidemiology. Clin Microbiol Rev. 2018;31(4) 10.1128/CMR.00020-18 PMC6148192 30209034

[29] Chen H (2021). Drivers of methicillin-resistant Staphylococcus aureus (MRSA) lineage replacement in China. Genome medicine.

[30] Jian Y (2021). Staphylococcus aureusIncreasing prevalence of hypervirulent ST5 methicillin susceptible subtype poses a serious clinical threat. Emerging microbes & infections.

[31] Aanensen D, et al. Whole-genome sequencing for routine pathogen surveillance in public health: a population snapshot of invasive Staphylococcus aureus in Europe. mBio. 2016;7(3) 10.1128/mBio.00444-16 PMC4959656 27150362

[32] Monecke S (2011). A field guide to pandemic, epidemic and sporadic clones of methicillin-resistant Staphylococcus aureus. PLoS One.

[33] Hong S, et al. qacAClinical and molecular characteristics of - and -positive methicillin-resistant causing bloodstream infections. Antimicrob Agents Chemother. 2019;63(4) 10.1128/AAC.02157-18 PMC6437476 30718251

[34] Cho O (2018). Prevalence and microbiological characteristics of qacA/B-positive methicillin-resistant Staphylococcus aureus isolates in a surgical intensive care unit. Microb Drug Resist.

[35] Smith J (2022). Genome evolution of invasive methicillin-resistant Staphylococcus aureus in the Americas. Microbiology spectrum.

[36] Kaku N (2022). Changing molecular epidemiology and characteristics of MRSA isolated from bloodstream infections: nationwide surveillance in Japan in 2019. J Antimicrob Chemother.

[37] Conceição T (2007). Replacement of methicillin-resistant Staphylococcus aureus clones in Hungary over time: a 10-year surveillance study. Clinical microbiology and infection : the official publication of the European Society of Clinical Microbiology and Infectious Diseases.

[38] Chen Y (2018). Using Core-genome multilocus sequence typing to monitor the changing epidemiology of methicillin-resistant Staphylococcus aureus in a teaching hospital. Clinical infectious diseases : an official publication of the Infectious Diseases Society of America.

[39] Xiao M (2013). National surveillance of methicillin-resistant Staphylococcus aureus in China highlights a still-evolving epidemiology with 15 novel emerging multilocus sequence types. J Clin Microbiol.

[40] Jin Y (2021). The genetic feature and virulence determinant of highly virulent community-associated MRSA ST338-SCCmec Vb in China. Emerging microbes & infections.

[41] Wu Y (2021). Surveillance of multidrug-resistant bacterial infections in non-adult patients - Zhejiang Province, China, 2014-2019. China CDC weekly.

[42] Maeda M (2016). Analysis of staphylococcal toxins and clinical outcomes of methicillin-resistant Staphylococcus aureus bacteremia. Biol Pharm Bull.

[43] Hu Q (2015). Panton-valentine leukocidin (PVL)-positive health care-associated methicillin-resistant Staphylococcus aureus isolates are associated with skin and soft tissue infections and colonized mainly by infective PVL-encoding bacteriophages. J Clin Microbiol.

[44] Wang X (2018). Staphylococcus aureusMolecular characteristics of community-associated isolates from pediatric patients with bloodstream infections between 2012 and 2017 in Shanghai, China. Front Microbiol.

