# Supplementary material for: High prevalence of ST5-SCCmec II-t311 clone of methicillin-resistant Staphylococcus aureus isolated from bloodstream infections in East China
Source: BMC Microbiol. 2024 Mar 16;24:89. doi: 10.1186/s12866-024-03232-5 (PMC10943896; doi:10.1186/s12866-024-03232-5)
Supplement: Supplementary file 1 — Supplementary material 1. [file 12866_2024_3232_MOESM1_ESM.docx]

Table S1 The primers sequences of virulence genes.

| Primer set and gene | Primer | Oligonucleotide sequence (5′-3′  ) | Size (bp) of PCR product |
| --- | --- | --- | --- |
| *sea* | Sea-F | CCTTTGGAAACGGTTAAAACG | 127 |
|  | Sea-R | TCTGAACCTTCCCATCAAAAAC |  |
| *seb* | Seb-F | TCGCATCAAACTGACAAACG | 477 |
|  | Seb-R | GCAGGTACTCTATAAGTGCCTGC |  |
| *sec* | Sec-F | CTCAAGAACTAGACATAAAAGCTAGG | 271 |
|  | Sec-R | TCAAAATCGGATTAACATTATCC |  |
| *sed* | Sed-F | CTAGTTTGGTAATATCTCCTTTAAACG | 319 |
|  | Sed-R | TTAATGCTATATCTTATAGGGTAAACATC |  |
| *see* | See-F | CAGTACCTATAGATAAAGTTAAAACAAGC | 178 |
|  | See-R | TAACTTACCGTGGACCCTTC |  |
| *seg* | Seg-F | AAGTAGACATTTTTGGCGTTCC | 287 |
|  | Seg-R | AGAACCATCAAACTCGTATAGC |  |
| *seh* | Seh-F | GTCTATATGGAGGTACAACACT | 213 |
|  | Seh-R | GACCTTTACTTATTTCGCTGTC |  |
| *sei* | Sei-F | GGTGATATTGGTGTAGGTAAC | 454 |
|  | Sei-R | ATCCATATTCTTTGCCTTTACCAG |  |
| *sej* | Sej-F | CAGCGATAGCAAAAATGAAACA | 426 |
|  | Sej-R | TCTAGCGGAACAACAGTTCTGA |  |
| *sel* | Sel-F | CATACAGTCTTACTAACGG | 275 |
|  | Sel-R | TTTTCTGCTTTAGTAACACC |  |
| *sem* | Sem-F | CTTGTCCTGTTCCAGTATC | 329 |
|  | Sem-R | ATACGGTGGAGTTACATTAG |  |
| *sen* | sen-F | ATTGTTCTACATAGCTGCAA | 682 |
|  | Sen-R | TTGAAAAAACTCTGCTCCCA |  |
| *seo* | Seo-F | AGTCAAGTGTAGACCCTATT | 534 |
|  | Seo-R | TATGCTCCGAATGAGAATGA |  |
| *sep* | Sep-F | GACCTTGGTTCAAAAGACACC | 275 |
|  | Sep-R | TGTCTTGACTGAAGGTCTAGC |  |
| *seq* | Seq-F | TCTAGCATATGCTGATGTAGG | 383 |
|  | Seq-R | CAATCTCTTGAGCAGTTACYTC |  |
| *fnbA* | FnbA-F | CACAACCAGCAAATATAG | 1279 |
|  | FnbA-R | CTGTGTGGTAATCAATGTC |  |
| *tsst1* | tsst1-F | AAGCCCTTTGTTGCTTGCG | 445 |
|  | tsst1-R | ATCGAACTTTGGCCCATACTTT |  |
| *eta* | Eta-F | CTAGTGCATTTGTTATTCAAGACG | 119 |
|  | Eta-R | TGCATTGACACCATAGTACTTATTC |  |
| *etb* | Etb-F | ACGGCTATATACATTCAATTCAATG | 262 |
|  | Etb-R | AAAGTTATTCATTTAATGCACTGTCTC |  |
| *Luk-PV* | Luk-PV-F | ATCATTAGGTAAAATGTCTGGACATGATCCA | 433 |
|  | Luk-PV-R | GCATCAACTGTATTGGATAGCAAAAGC |  |
| *lukDE* | LukDE-F | TTAGGATATAACATTGGAGGTA | 1499 |
|  | LukDE-R | GATTAGTTTCTTTAGAATCCGT |  |
| *hla* | Hla-F | TTT TCA GGG TCA ATA TAA GC | 209 |
|  | Hla-R | CTTTCCAGCCTACTTTTTTATCAGT |  |
| *hlb* | Hlb-F | GTGCACTTACTGACAATAGTGC | 309 |
|  | Hla-R | GTTGATGAGTAGCTACCTTCAGT |  |
| *hlg* | Hlg-F | TTGGCTGGGGAGTTGAAGCACA | 306 |
|  | Hlg-R | CGCCTGCCCAGTAGAAGCCATT |  |
| *hly* | Hly-F | TGCAAGTCCTAAGACGCCAA | 753 |
|  | Hly-R | CCACACTTGAGATATATGCAGGA |  |
| *icaA* | IcaA-F | CCTAACTAACGAAAGGTAG | 1315 |
|  | IcaA-R | AAGATATAGCGATAAGTGC |  |
| *clfA* | ClfA-F | GCTTCAGTGCTTGT AGG | 980 |
|  | ClfA-R | TTT TCA GGG TCA ATA TAA GC |  |
| *sdrC* | SdrC-F | CGCATGGCAGTGAATACTGTTGCAGC | 731 |
|  | SdrC-R | GAAGTATCAGGGGTGAAACTATCCACAAATTG |  |
| *sdrD* | SdrD-F | GGAAATAAAGTTGAAGTTTC | 500 |
|  | SdrD-R | ACTTTGTCATCAACTGTAAT |  |
| *sdrE* | SdrE-F | CAGTAAATGTGTCAAAAGA | 767 |
|  | SdrE-R | TTGACTACCAGCTATATC |  |

2.The primers sequences of MLST, Spa and *SCCmec* typing :

arc up - 5' TTG ATT CAC CAG CGC GTA TTG TC -3'

arc dn - 5' AGG TAT CTG CTT CAA TCA GCG -3'

aro up - 5' ATC GGA AAT CCT ATT TCA CAT TC -3'

aro dn - 5' GGT GTT GTA TTA ATA ACG ATA TC -3'

glp up - 5' CTA GGA ACT GCA ATC TTA ATC C -3'

glp dn - 5' TGG TAA AAT CGC ATG TCC AAT TC -3'

gmk up - 5' ATC GTT TTA TCG GGA CCA TC -3'

gmk dn - 5' TCA TTA ACT ACA ACG TAA TCG TA -3'

pta up - 5' GTT AAA ATC GTA TTA CCT GAA GG -3'

pta dn - 5' GAC CCT TTT GTT GAA AAG CTT AA -3'

tpi up - 5' TCG TTC ATT CTG AAC GTC GTG AA -3'

tpi dn - 5' TTT GCA CCT TCT AAC AAT TGT AC -3'

yqi up- 5' CAG CAT ACA GGA CAC CTA TTG GC -3'

yqi dn- 5' CGT TGA GGA ATC GAT ACT GGA AC -3'

Spa up5′-GACGATCCTTCAGTGAGCAAAG-3′

Spa dn5′-GCAGCAATTTTGTCAGCAGTAG-3′

SCCmec I up5′-GCTTTAAAGAGTGTCGTTACAGG-3′

SCCmec I dn5′-GTTCTCTCATAGTATGACGTCC-3′

SCCmec II up5′-CGTTGAAGATGATGAAGCG-3′

SCCmec II dn5′-CGAAATCAATGGTTAATGGACC-3′

SCCmec III up5′-CCATATTGTGTACGATGCG-3′

SCCmec III up5′-CCTTAGTTGTCGTAACAGATCG-3′

SCCmec IV up5′-GCCTTATTCGAAGAAACCG-3′

SCCmec IV dn5′-CTACTCTTCTGAAAAGCGTCG-3′

SCCmec V up5′-GAACATTGTTACTTAAATGAGCG-3′

SCCmec V dn5′-TGAAAGTTGTACCCTTGACACC-3′

mecA up5′-GTG AAG ATA TAC CAA GTG ATT-3′

mecA dn5′-ATG CGC TAT AGA TTG AAA GGA T-3′

4.The PCR related steps and parameters: The amplification was performed in a GeneAmp PCR system 9700 or 9600 Thermal Cycler (Applied Biosystems, Foster City, CA) with an initial denaturation step at 95℃ for 5 min, 35 cycles of denaturation 95℃ for 1 min, annealing 55-60℃ for 1 min,extension 72℃ for 1 min, final extension 72℃ for 10 min and followed by a hold at 4℃. Each PCR experiment was carried out with positive control and negative control (distilled water). The primer annealing temperature was appropriately adjusted according to the primer conditions.
