# Supplementary material for: High prevalence of ST5-SCCmec II-t311 clone of methicillin-resistant Staphylococcus aureus isolated from bloodstream infections in East China
Source: BMC Microbiol. 2024 Mar 16;24:89. doi: 10.1186/s12866-024-03232-5 (PMC10943896; doi:10.1186/s12866-024-03232-5)
Supplement: Supplementary file 2 — Supplementary material 2. [file 12866_2024_3232_MOESM2_ESM.docx]

**Table S2. The antibiotic resistance rates of MRSA ST5 and ST59 isolates.**

| **Antibiotics^a^** | **Total**  **(n^b^=263); R^c^** | **ST5**  **(n=131); R** | **ST59**  **(n=44); R** |
| --- | --- | --- | --- |
| OXA | 263; 100.0% | 131; 100.0% | 44; 100.0% |
| PEN | 263; 100.0% | 131; 100.0% | 44; 100.0% |
| ERY | 214; 81.4% | 123; 93.9% | 31; 71.5% |
| CLN | 123; 46.8% | 53; 40.5% | 29; 65.9% |
| SXT | 4; 1.5% | 1; 0.8% | 0; 0% |
| TCY | 97; 36.9% | 68; 51.9% | 10; 22.7% |
| VAN | 0; 0.0% | 0; 0.0% | 0; 0% |
| RIF | 3; 1.4% | 2; 1.5% | 0; 0% |
| CIP | 150; 57.0% | 100; 76.3% | 15; 34.1% |
| LVX | 168; 63.9% | 123; 93.9% | 9; 20.5% |
| MFX | 161; 61.2% | 124; 94.7% | 6; 13.6% |
| AMK | 2; 0.8% | 1; 0.8% | 1; 2.3% |
| TGC | 9; 3.4% | 7; 5.3% | 1; 2.3% |
| LNZ | 0; 0.0% | 0; 0% | 0; 0% |
| DAP | 0; 0.0% | 0; 0% | 0; 0% |

^a^Oxacillin (OXA), penicillin (PEN), erythromycin (ERY), clindamycin (CLN), trimethoprim-sulfamethoxazole (SXT), tetracycline (TCY), vancomycin (VAN), rifampicin (RIF), ciprofloxacin (CIP), levofloxacin (LVX), moxifloxacin (MFX), amikacin (AMK), tigecycline (TGC), linezolid (LNZ), daptomycin (DAP).

^b^n, number of isolates in each type.

^c^R = resistance.
