# Supplementary material for: High prevalence of ST5-SCCmec II-t311 clone of methicillin-resistant Staphylococcus aureus isolated from bloodstream infections in East China
Source: BMC Microbiol. 2024 Mar 16;24:89. doi: 10.1186/s12866-024-03232-5 (PMC10943896; doi:10.1186/s12866-024-03232-5)
Supplement: Supplementary file 3 — Supplementary material 3. [file 12866_2024_3232_MOESM3_ESM.docx]

**Table S3.The proportion of MRSA ST5-II-t311 isolates from 2014 to 2019.**

| **Year** | **Total** | **The number of ST5-II-t311 isolates (n=110)** |
| --- | --- | --- |
| 2014 | 33 | 21 |
| 2015 | 51 | 30 |
| 2016 | 46 | 20 |
| 2017 | 38 | 15 |
| 2018 | 46 | 13 |
| 2019 | 49 | 11 |
